# Pharmaceutical Benefits of Fluticasone Propionate Association to Delivery Systems: In Vitro and In Vivo Evaluation

**DOI:** 10.3390/pharmaceutics11100521

**Published:** 2019-10-10

**Authors:** Marina G. Dogbe, Ambinintsoa Yattussia Mafilaza, Carla Vânia Eleutério, Helena Cabral-Marques, Sandra Simões, Maria Manuela Gaspar

**Affiliations:** 1 Institut des Sciences Pharmaceutiques et Biologiques, Faculté de Pharmacie de Lyon, 92, Rue Pasteur, 69,361 Lyon, France; madogbe@yahoo.fr (M.G.D.); yattuw@yahoo.fr (A.Y.M.); 2 iMedUlisboa, Faculty of Pharmacy, Universidade de Lisboa, Av. Prof. Gama Pinto, 1649-003 Lisboa, Portugal; carlavania@ff.ulisboa.pt (C.V.E.); hcmarques@ff.ulisboa.pt (H.C.-M.)

**Keywords:** fluticasone propionate, pulmonary delivery, intranasal delivery, liposomes, cyclodextrins, in vivo studies, asthma murine model

## Abstract

The objective of the present work was to characterize the ability of liposomes and cyclodextrin (CyD) complexes to modulate the in vivo profile of fluticasone (FTZ). In vitro cell compatibility tests were performed, exposing A549 cells to FTZ in the free form and FTZ associated to liposomes and complexed with CyD. The in vivo fate of a selected FTZ liposomal formulation and of several FTZ CyD complexes was achieved following intranasal instillation or pulmonary administration in BALB/c mice, respectively. For pulmonary administration, an inhalation chamber was constructed to enable the simultaneously pulmonary administration to six mice. Thirty minutes and 3 h after administration, mice were sacrificed, their blood, lungs, livers, and spleens were removed, and FTZ level was determined by HPLC using an extraction procedure. The in vitro tests revealed no toxic effects of FTZ formulations, as cellular viability was always superior to 90% for FTZ concentrations ranging from 5 to 60 µM 72 h after incubation. The in vivo biodistribution results showed that FTZ incorporated in liposomes resulted in 20 and 30 times higher accumulation in the lungs in comparison with free FTZ, at 0.5 and 3 h after i.n. administration, respectively. FTZ associated to Hydroxypropyl-γ-cyclodextrin (HP-CyD) was the complex that permitted the higher accumulation of FTZ in the lungs in comparison with the respective free form. The results also suggest that the inhalation chamber apparatus can effectively facilitate the evaluation of in vivo inhalation. The establishment of an animal model of asthma allows us to further study the therapeutic efficacy of the developed FTZ formulations.

## 1. Background

Allergic rhinitis and bronchial asthma are airway inflammation chronic diseases that often coexist [1]. Moreover, allergic rhinitis is a common comorbidity of asthma that contributes to asthma severity [2]. As a result of the co-existence of asthma and allergic rhinitis, targeting both pathologies represent a strategy for the development of new joint therapies [3,4,5].

Currently, therapy consists of controlling symptoms and minimizing associated risks. Bronchodilators (short-, long-, and ultra-long acting) and anti-inflammatory drugs such as glucocorticoids (GCS), leukotriene-receptor antagonists, and theophylline are some of the existing drugs on the market.

Among GCS, fluticasone propionate (FTZ) is one of the drugs delivered in a dry powder inhaler or by nasal instillation. Nasal and pulmonary delivery routes of administration are non-invasive, convenient, and comfortable, aiming the direct delivery of FTZ to the lungs [5].

Several strategies for improving pulmonary or nasal administration of low or high molecular weight molecules have been widely described [6,7]. In the present work, and using FTZ as a model drug relevant for pulmonary administration [8], two strategies were addressed following its incorporation in liposomes or in Cyclodextrin (CyD) complexes. Table 1 contains some physical properties of this molecule, namely chemical structure, molecular weight, octanol/water partition coefficient (LogP), and solubility in water.

Liposomes are undeniable biocompatible lipid systems able to increase the solubility of many lipophilic drugs, such as the case of FTZ [9,10,11,12,13,14] while improving delivery properties across nasal mucosa by promoting a prolonged contact between the drug and the absorptive sites in the nasal cavity [15]. On the other hand, CyD are versatile excipients with great potential as drug carriers in pharmaceutical technology able to improve solubility, bioavailability, and stability of associated drugs [16], particularly for pulmonary delivery [17,18]. 

In this work, liposomal formulations of FTZ were developed for further intranasal administration, while FTZ cyclodextrin (CyD) complexes previously developed [17] were tested for pulmonary administration as dry powders. All FTZ formulations were submitted to in vitro studies in the presence of a respiratory epithelial cell line, the A549, to evaluate their influence on cell viability. The biodistribution profile of the two FTZ delivery systems was performed aiming to compare the lung accumulation levels of the glucocorticoid using liposomes and CyD complexes following intranasal and pulmonary delivery, respectively. Particularly, for the pulmonary delivery of FTZ CyD complexes, a nose-only chamber was designed, enabling the simultaneous administration for six mice [19].

## 2. Materials and Methods

### 2.1. Materials

Fluticasone propionate (FTZ) was a kind gift from Hovione Farmacêutica, S.A. (Loures, Portugal). The CyDs used, Acetyl-γ-cyclodextrin (Acetyl-γ-CyD), was a gift from Cyclolab (Budapest, Hungary), Hydroxypropyl-γ-cyclodextrin (HP-γ-CyD) and γ-cyclodextrin (γ-CyD) were a gift from Wacker (Burghauser, Germany). Pure phospholipids, egg phosphatidyl choline (PC), and phosphatidyl glycerol (PG) were purchased from Avanti Polar Lipids (Alabaster, AL, USA). Cholesterol (CHOL), stearylamine (SA), Sodium dodecyl sulphate (SDS), dimethylsulfoxide (DMSO), Tween^®^ 80, and phosphate buffered saline (PBS) were obtained from Sigma (St. Louis, MO, USA). Polycarbonate membranes from Nucleopore Track-Etched were purchased from Whatman, Ltd. (Clifton, NJ, USA). Dulbecco’s Modified Eagle Medium (DMEM), fetal bovine serum (FBS), TrypLE™ Express, penicilin/streptomycin, and Trypan Blue from Gibco were obtained from Invitrogen, Life Technologies Corporation (Grand Island, NY, USA). Reagents for in vitro assays were obtained from Promega (Madison, WI, USA). 

Acetonitrile (HPLC grade) was from Merck (Darmstadt, Germany). Highly purified water was of Milli-Q quality and obtained from ELIX-3 equipment (Merck Millipore, Darmstadt, Germany). All other reagents and solvents were of the purest grade available, and generally were used without further treatment. 

### 2.2. Cell Lines 

The human alveolar adenocarcinoma cell line, the A549 line (ATCC^®^ CCL-185™), was obtained from LGC Standards, Barcelona, Spain. 

### 2.3. Animals

Male BALB/c mice were obtained from Gulbenkian Institute of Science (Oeiras, Portugal). Animals were kept on an animal room under standard hygiene conditions, with access to commercial chow and acidified drinking water ad libitum. All animal experiments were conducted according to the animal welfare organ of the Faculty of Pharmacy, Universidade de Lisboa, approved by the competent national authority Direção Geral de Alimentação e Veterinária (DGAV) on the 22/09/2016, (Project “Lung CYD-DDS (cyclodextrin-based drug delivery systems for inhalation”) and in accordance with the EU Directive (2010/63/UE) and Portuguese laws (DL 113/2013, 2880/2015, 260/2016, and 1/2019).

### 2.4. Preparation of Fluticasone Propionate (FTZ) Liposomes

Liposomes composed of the selected phospholipids were prepared by the de-hydration re-hydration method (DRV) [21]. To resume, the selected phospholipids (20 µmol/mL) were mixed with FTZ at 500 µg/mL and solubilized in chloroform. The mixture was dried in a rotary evaporator (Buchi, Switzerland) until achievement of a thin lipid film in a round flask.

This film was then dispersed in deionized water and the liposomal suspension was frozen (−70 °C) and lyophilized in a freeze dryer (Edwards, CO, USA) overnight. 

The re-hydration of the lyophilized powder was performed with hydroxyethyl piperazineethane sulfonic acid (HEPES) buffer (10 mM HEPES, 145 mM NaCl, pH 7.4) in two steps, in order to enhance FTZ incorporation. In the first hydration step, for 30 min, two-tenths of the volume of the original dispersion was added and subsequently the addition of HEPES buffer, up to the starting volume, was made. In order to reduce and homogenize the mean size of liposomes, the so-formed suspensions were submitted to an extrusion step through polycarbonate membranes of appropriate pore sizes until the desired mean size of liposomes was reached (0.8, 0.6, 0.4, 0.2 µm) under nitrogen pressure (10−500 1b/in^2^) with an Extruder device Lipex (Biomembranes Inc., Vancouver, BC, Canada). It is important to refer that the re-hydration and extrusion steps were always performed at a temperature above the transition temperature (*T*c) of the phospholipids.

The separation of non-incorporated FTZ was performed by gel filtration (Econo-Pac^®^ 10DG, Bio-Rad Laboratories, Hercules, CA, USA) followed by ultracentrifugation at 250,000 *g* for 120 min, at 15 °C, in a Beckman LM-80 ultracentrifuge (Beckman Instruments, Inc., Palo Alto, CA, USA). At the end, the pellet was suspended in HEPES buffer, according to the final concentration desired for FTZ liposomes. Unloaded liposomes, used in in vitro tests, were also performed for all lipid compositions tested. After rotary evaporator, the lipid film was dispersed in HEPES buffer and the resultant suspensions were extruded as above referred for FTZ loaded liposomes. 

### 2.5. Characterization of FTZ Liposomes

The liposomal formulations were characterized in terms of lipid composition, lipids and FTZ concentrations, mean size, and zeta potential. The FTZ quantification either in liposomes or in organs was performed by HPLC (Section 2.7), according to Couto et al. (2014) [18], with some modifications. The lipid content of the formulations was determined using a spectrophotometric technique [22]. Unloaded liposomes were characterized in terms of lipid content, mean size, and zeta potential.

#### 2.5.1. Incorporation Efficiency 

After quantification of lipids and FTZ, the incorporation efficiency (I.E.) was calculated using the following Equation:

I.E. (%) = [(FTZ/Lip)_f_/(FTZ/Lip)_i_] × 100

where: (FTZ/Lip)_f_ means Final FTZ to lipid ratio; (FTZ/Lip)_i_ means initial FTZ to lipid ratio; I.E. is a measure of the efficiency of a lipid mixture for incorporating FTZ.

#### 2.5.2. Liposome Size Measurements

Liposome mean diameter was determined by dynamic light scattering in a hydrodynamic sizing system (Zetasizer Nano S, Malvern Instruments, Malvern, UK). As a measure of particle size distribution of liposomes, the system reports the polydispersity index (PdI). The index ranges from 0.0 for an entirely monodisperse sample up to 1.0 for a polydisperse suspension. Liposomal formulations were previously diluted (1:100) in HEPES buffer. To ensure that appropriate mean diameter and PdI were achieved, size measurements were performed during the extrusion step and in final formulations. Each analysis was carried out at 25 °C in triplicate.

#### 2.5.3. Zeta Potential Determination

Zeta potential of liposomal preparation was measured by Laser Doppler Electrophoresis (Zetasizer Nano Z, Malvern instruments, UK). Liposomal formulations were previously diluted (1:100) in HEPES buffer. The zeta potential of samples was recorded at a temperature of 25 °C, in triplicate.

### 2.6. Preparation and Characterization of FTZ Cyclodextrin Complexes

FTZ was formulated in different CyDs (Table 3) and resulting dry powders were characterized according to the method described in Drumond et al. (2014) [17]. For in vitro studies, CyD complex dry powders were prepared using the same methodology, but without FTZ. 

### 2.7. Chromatographic System for FTZ Quantification

The HPLC used was a System Gold (Beckman Instruments, Inc., USA) constituted by a 168 Diode-Array Detector, a 126 Solvent Module, and a Midas Spark 1.1 auto-sampler (Spark, Netherlands). The analytical column was a LiChrospher^®^ 100 (125 × 4.0 mm) RP-18 (5 µm), (Merck, Darmstadt, Germany). The apparatus was connected to a desk computer with the 32 KARAT Software (Beckman Coulter, USA) for integration and treatment of chromatograms. 

The mobile phase consisted of acetonitrile (ACN) and water (60:40, *v*/*v*) with a flow rate of 1.0 mL/min. FTZ analysis was performed by UV detection at a fixed wavelength of 236 nm and the loop was of 10 µL. 

#### Preparation of Standard Solutions

Stock solutions of FTZ at 200 µg/mL in ACN were prepared. FTZ standards ranging from 2 to 40 µg/mL were made by diluting the respective initial stock solution with ACN. Samples from liposomes and after extraction in organs were diluted in ACN in the range of calibration curves. Standards and samples were filtered through 0.2 μm porosity polytetrafluoroethylene (PTFE) membranes before injection onto HPLC. An intermediate standard was always injected together with the analyzed samples to verify the precision of the obtained chromatograms from their peak area and concentration response.

For determining the concentration average of FTZ in each CyD complex, 5 mg of each FTZ CyD complex was weighted, diluted in ACN, and filtered through 0.2 µm porosity PTFE membranes before injection onto HPLC. 

### 2.8. In Vitro Experiments 

#### 2.8.1. Cell Culture Maintenance

The A549 cell line was grown in culture flasks and maintained in DMEM, supplemented with 10% (*v*/*v*) of heat inactivated FBS (iFBS) and 1% (*v*/*v*) penicillin/streptomycin in a humidified atmospheric incubator at 37 °C, 5% CO_2_ [23]. The maintenance of cultures was performed every three to four days, until cells reached a confluence of about 80%. At this point, sub-culturing was performed using a solution of TripLE^TM^ Express. Briefly, after media removal, the cell layer was washed with PBS and incubated with TripLE^TM^ Express for 7–10 min at 37 °C. After cells’ detachment, complete medium was added. The cells were then centrifuged in a bench centrifuge (Beckman, Izasa, Spain) at 500× *g* for 10 min and the pellet was suspended in fresh medium. Appropriate aliquots of the cell suspension were seeded in new culture flasks. 

The storage of cell lines was done in liquid nitrogen with freezing medium consisting of iFBS and 10% (*v*/*v*) of DMSO.

#### 2.8.2. Cellular Viability Assay 

Cell viability was evaluated in the absence or presence of increasing concentrations of FTZ in free and liposomal forms as well as incorporated in CyD complexes, by measuring mitochondrial activity, based on the colorimetric method, the MTS (3-(4,5-dimethylthiazol-2-yl)-5-(3-carboxymethoxyphenyl)-2-(4-sulfophenyl)-2*H*-tetrazolium), from Promega (Madison, WI, USA) [23]. Briefly, dehydrogenase enzymes found in metabolically active cells convert tetrazolium compound to a water-soluble formazan dye, which can be quantified spectrophotometrically at λ = 490 nm, being the absorbance directly correlated to viable cells [23].

A549 cells at a concentration of 5 × 10^4^/mL were plated into 96-well plates (200 µL) under the culture conditions described before. Twenty-four hours after plating, medium was removed, and adherent cells were incubated with FTZ in free or incorporated in liposomes or in CyD complexes at a concentration ranging from 5 to 60 µM. Negative and positive controls were also used in parallel. Negative control was constituted by cells in the presence of complete medium. The positive control was constituted by 0.2% (*w*/*v*) SDS. In addition, as the stock free FTZ solution was prepared in DMSO, cells were also incubated with DMSO at 0.24% (*v*/*v*) corresponding to the highest concentration used for FTZ solubilization. Unloaded liposomes and CyD complexes constituted other controls, using the same lipid concentrations as in FTZ liposomes and the same amount as the one used in the respective FTZ CyD complex, respectively. All tests were performed in sextuplicate for each tested concentration and controls. After 72 h of incubation, culture medium was removed from all wells, and replaced with 100 µL of incomplete medium and 20 µL, of the combined PMS/MTS solution (1:20 *v*/*v*) freshly prepared, into each well according to manufacturer’s instructions. Plates were slightly agitated and incubated for 60 min, under the same culture conditions mentioned before. 

Absorbance was measured at λ = 490 nm (Biotek Instruments^®^, Winooski, VT, USA). The absorbance of negative control (cells incubated with complete medium) was set as 100% viability. 

A triplicate set of control wells (without cells, Blank) containing the same volumes of culture medium and combined PMS/MTS solution as in the experimental wells was also prepared. The absorbance average at λ = 490 nm from these Blank control wells was subtracted from all other absorbance values to yield corrected absorbances.

The cellular viability (%) was calculated as follows:

$$\mathrm{Cellular Viability}(\%)=\frac{A-B}{M-B}\times 100$$

where *A* is the absorbance obtained for each concentrations of the tested formulation; *B* is the absorbance average obtained for the Blank; *M* is the absorbance average for negative control (cells incubated with complete medium).

### 2.9. Biodistribution Studies of FTZ Formulations

Biodistribution studies were performed in healthy BALB/C mice after intranasal (i.n.) administration of FTZ in free or liposomal forms at a dose of 1 mg/kg of body weight (40 µL/nostril).

**Figure 1:**
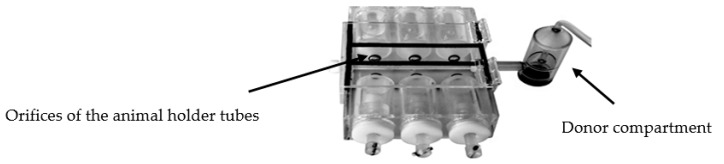
Photograph of the inhalation chamber [19].

For the biodistribution studies of FTZ CyD complexes the formulations were pulmonary administered using a nose-only chamber that simultaneously allows the exposition of six animals to the dry powders [19]. Mice were restrained with their nostrils inserted snugly into the inhalation chamber (Figure 1). About 200 mg of CyD formulations were placed into the donor compartment of the apparatus. To prepare free FTZ in order to obtain the same amount of FTZ per gram of powder, FTZ was diluted with lactose of analytical grade (Fagron, Barcelona, Spain). The dry powders of all FTZ formulations were then dispersed by an airflow generated under an air pressure of 0.4 kg/cm^2^ for 5 min. 

Half an hour and three hours after i.n. administration or inhalation exposure, mice were sacrificed, blood was collected into heparinized tubes, and stored at −30 °C. Lung, spleen, and liver were removed and stored at −70 °C, until extraction procedure. At least three animals were used for each analyzed time, for each formulation tested.

#### FTZ Extraction from Tissues 

FTZ levels in tissues were determined by HPLC after an extraction procedure according to Gaspar et al. [10]. Liver, lung, and spleen tissues were thawed and aliquots of ca. 100 mg were weighed out for each sample and extracted twice with 2300 µL of dichloromethane: Isooctane mixture (2:3, *v*/*v*) by mechanical shaking, for 30 min at room temperature, followed by a centrifugation step at 1200× *g* for 10 min in a bench centrifuge (Beckman, Izasa, Spain). All the organic extracts were pooled and evaporated to dryness under nitrogen. The residue was dissolved in 500 µL of ACN filtered through 0.2 μm porosity PTFE membranes and then injected onto the HPLC system. To determine the efficiency of the extraction procedures, a known amount of FTZ was added to organs removed from mice that had not received FTZ and then submitted to the same above-mentioned extraction protocol. 

### 2.10. Murine Model of Asthma

**Figure 2:**
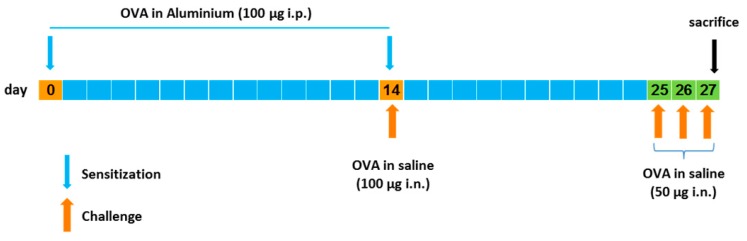
Schematic representation of the experimental allergic lung inflammation induction in mice as performed in this study.

The allergic lung inflammation in mice was induced according to Henderson et al. [24] and Oliveira et al. with some modifications [25]. In Figure 2, a schematic representation of the lung inflammation model used is shown. Briefly, mice were sensitized on days 0 and 14 by receiving intraperitoneal (i.p.) injections of 0.2 mL (100 µg) of chicken ovalbumin (OVA) in aluminum hydroxide (10 mg/mL). On days 14, 25, 26, and 27, mice were challenged with intranasal (i.n.) administrations of OVA in saline: 100 µg on day 14 and 50 µg on days 25, 26, and 27. Mice were sacrificed one day after the last OVA challenge and their lungs were removed and fixed in 10% neutral formalin. Lung sections were stained with hematoxylin and eosin for histologic analysis. 

## 3. Results

### 3.1. FTZ Liposomes

The method selected for the preparation of FTZ liposomes was the DRV method followed by an extrusion step [21,26]. Taking into account the hydrophobic character of FTZ (Table 1), it is expected that it will accommodate in the lipid bilayer and so the phospholipids selected were neutral and negatively charged, with a low phase transition temperature (−6 °C): Phosphatidyl choline (PC) and phosphatidyl glycerol (PG), respectively. The influence of the presence in the lipid composition of a positively charged surfactant, stearylamine (SA), or cholesterol (CHOL), on FTZ incorporation parameters was also evaluated. The physicochemical characteristics for all the selected lipid mixtures in terms of particle size, zeta potential, and FTZ incorporation parameters are reported in Table 2. 

All tested liposomal formulations presented mean sizes ranging from 0.16 to 0.21 µm, with a PdI below 0.2, evidencing the high homogeneity of all prepared nanoformulations. The zeta potential of FTZ liposomes was dependent on the lipid composition. The presence of PG or SA resulted in negatively or positively charged nanoformulations with a zeta potential of −25 and +5 mV, respectively. PC liposomes in the absence or presence of CHOL presented zeta potential values close to neutrality. The incorporation efficiency of the FTZ was lipid composition dependent with higher values for neutral or negatively charged nanoformulations. The inclusion of CHOL led to a decrease on incorporation parameters. When included in the lipid composition of liposomes, CHOL is inserted within bilayers competing with the accommodation of hydrophobic molecules such as FTZ. This effect is in accordance with the literature [23,27,28,29].

### 3.2. FTZ Cyclodextrin Complexes

FTZ was also formulated in different CyDs as dry powders. The chemical structure and molecular weight of the CyDs used, as well as the mass median aerodynamic diameter (MMAD) of the FTZ CyD complexes previously prepared [17] and tested in the present work, are shown in Table 3. In addition, the particles distribution in percentage was also included. For example, the d95 and d5 obtained for the FTZ CyD complexes mean that 95% of the particles presented a mean size below 5.98 to 6.81 µm and only 5% of the particles are below 1.17 µm. These results confirm the suitability of developed FTZ CyD complexes for pulmonary delivery [30].

The HPLC represents a widely used technique for qualitative and quantitative analysis of different kind of molecules. The method used in the present work [18] allowed the quantification of FTZ incorporated in CyDs, after previous disruption with ACN, as well as the amount of the glucocorticoid in different organs after BALB/c mice exposition to dry powder FTZ formulations [18]. Table 4 shows the amount of FTZ incorporated in the different CyD complexes. 

### 3.3. In Vitro Studies

**Figure 3:**
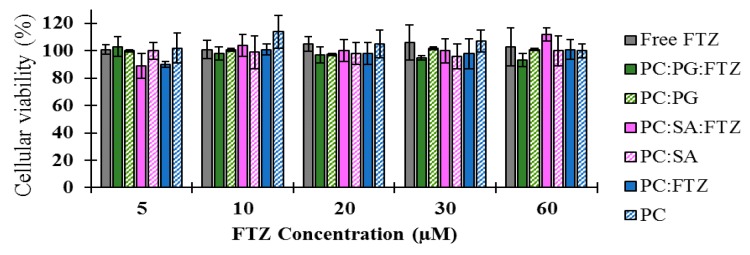
Cellular viability of A549 cells, 72 h after incubation with FTZ in the free form (Free FTZ), incorporated in liposomes [PC:PG:FTZ (N4, Table 2); PC:SA:FTZ (N3, Table 2); PC:FTZ (N1, Table 2)] or in unloaded liposomes (PC:PG; PC:SA; PC). FTZ tested concentrations ranged from 5 to 60 µM. Unloaded liposomes were also used as control using the same lipid concentrations as the ones used for the respective FTZ liposomal formulation. The obtained results are expressed as a mean ± SD of sextuplicate values for each concentration and formulation tested.

**Figure 4:**
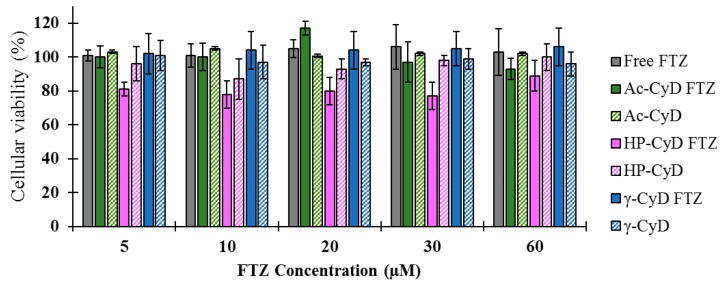
Cellular viability of A549 cells, 72 h after incubation with FTZ in the free form (Free FTZ), incorporated in CyD (Ac-CyD FTZ; HP-CyD FTZ; ϒ-CyD FTZ) or in unloaded CyD (Ac-CyD; HP-CyD; ϒ-CyD). FTZ tested concentrations ranged from 5 to 60 µM. Unloaded CyD were also used as control using the same amount as the ones used for the respective FTZ CyD complex. The obtained results are expressed as a mean ± SD of sextuplicate values for each concentration and formulation tested.

The human alveolar cell line, A549, was used to evaluate the biocompatibility of different formulations of FTZ either in the free form or incorporated in liposomes or in CyD complexes. FTZ was tested in a range of concentrations from 5 to 60 µM. Unloaded liposomes or unloaded CyD were also included using the same range of lipid concentrations or the same amount of CyD, respectively. The cellular viability, in percentage, of A549 cell line for all analyzed formulations is shown in Figure 3 for FTZ liposomal formulations and in Figure 4 for FTZ CyD complexes. Data was calculated in relation to negative control (cells incubated with complete medium) which corresponded to 100% of cellular viability. Regarding the data presented in Figure 2, the viability was always superior to 90% independent of the formulation tested, proving that FTZ and the phospholipids used for its incorporation were not toxic to the alveolar cell line. As the stock solution of FTZ was prepared in DMSO, a sample using the highest percentage used for FTZ solubilization (0.24% in complete medium) was also included in these assays. The viability was 100% related to control, proving that the maximum amount of DMSO was not interfering in cells viability. A positive control, SDS at 0.2% in complete medium, was also included. The cellular viability of this sample was below 1%.

According to the data presented in Figure 3, the viability of A549 cells was close to 100% related to the control. For the HP-CyD FTZ complex, a slight reduction (<20%) was observed, but only for the lowest concentrations tested. 

### 3.4. In Vivo Studies

#### 3.4.1. In Vivo Fate of FTZ Formulations

As mentioned before, the two delivery systems incorporating FTZ were designed to be tested in vivo using two different routes of administration: FTZ liposomes for i.n. administration and dry powders of FTZ CyD complexes for pulmonary delivery. 

**Figure 5:**
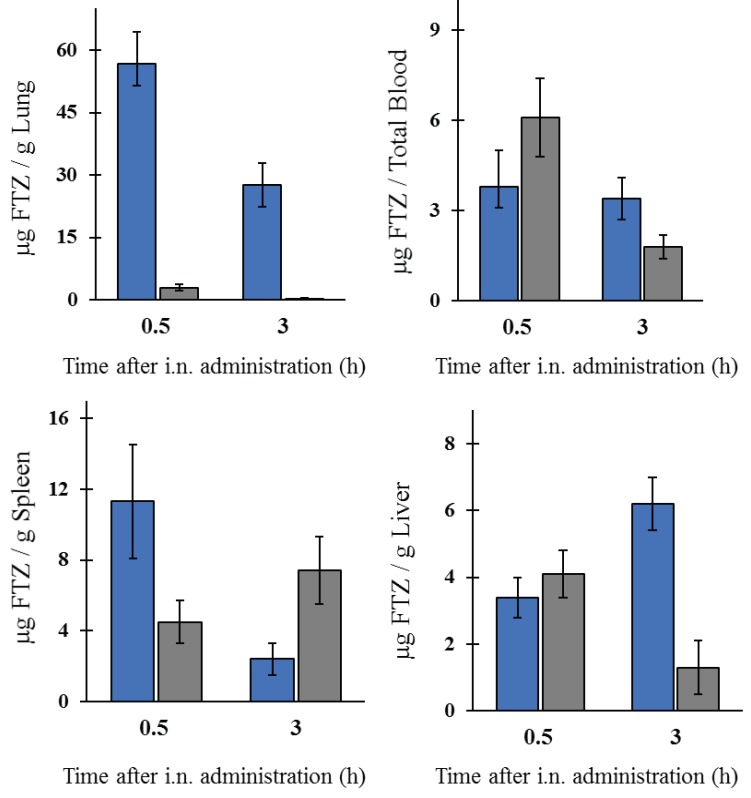
Biodistribution profile of FTZ in the free form (grey columns) or incorporated in liposomes (blue columns) after i.n. administration. FTZ levels in lung, blood, spleen, and liver. The obtained results are expressed as a mean ± SD of three animals per analyzed time and formulation. Mice received the formulation and were sacrificed 0.5 or 3 h after i.n. administration. The obtained results are expressed as mean ± SD (*n* = three mice per selected time and per FTZ formulation).

In the case of FTZ liposomes, the nanoformulation 1 (Table 2) was selected for in vivo assays. This liposomal nanoformulation showed the highest loading with a final (FTZ/Lip) ratio of 7 µg per µmol of lipid in comparison to the other nanoformulations prepared. Biodistribution studies were performed in healthy BALB/c mice after i.n. administration of FTZ in free or liposomal forms at a dose of 1 mg/kg of body weight. Two time points were chosen: 0.5 and 3 h after i.n. administration. At selected times mice were sacrificed, and blood, liver, spleen, and lungs were collected. Using an extraction protocol, FTZ was determined by HPLC. Obtained results are shown in Figure 5. 

The i.n. administration of FTZ in liposomal form led to much higher levels of the glucocorticoid in the lung, when compared to the respective free form. In fact, 0.5 h after instillation, the values obtained were 57 and 3 µg FTZ per gram of lung, respectively. In addition, 3 h after i.n. administration, mice that received FTZ liposomes still preserved high values of the glucocorticoid (28 µg per gram of organ), while for mice receiving the free drug, the value was below 1 µg. For the other analyzed organs, the differences observed were not so evident. For mice receiving liposomal formulation of the glucocorticoid, the accumulation of FTZ in the lungs clearly demonstrated the advantage regarding the use of the selected lipid-based delivery system.

**Figure 6:**
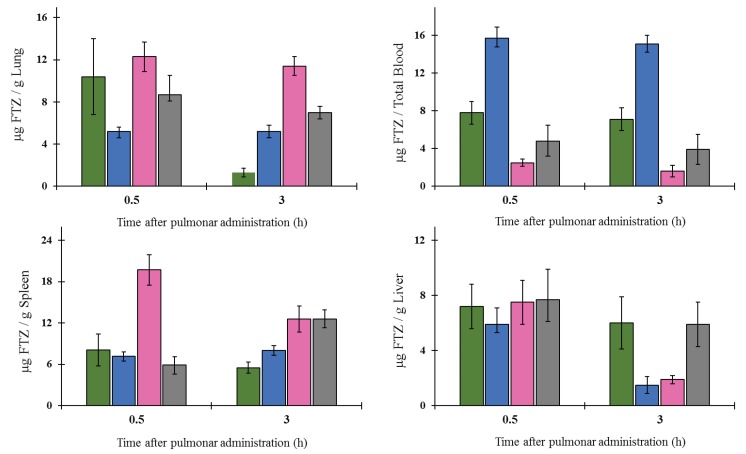
Biodistribution profile of FTZ in the lung, blood, spleen, and in the liver after inhalation of FTZ in free form (grey columns) or incorporated in Ac-CyD (green columns), γ-CyD (blue columns), or in HP-CyD (pink columns). Mice were exposed to formulations for 5 min and were sacrificed 0.5 or 3 h after pulmonary delivery of dry powders under study. The obtained results are expressed as mean ± SD (*n* = three mice per selected time and per FTZ formulation).

For the pulmonary delivery of dry powders of FTZ in the free form or incorporated in CyD complexes, the inhalation chamber (shown in Methods section) enabled the simultaneous pulmonary administration for six mice [19]. BALB/c mice were exposed during 5 min to dry powder formulations. In this in vivo assay, the three FTZ CyD complexes were tested and compared to FTZ in the free form. The amount of FTZ per gram of organ, 0.5 and 3 h after inhalation exposure, for tested formulations, is presented in Figure 6. 

According to the results obtained, the inhalation of FTZ HP-CyD complex was able to maintain FTZ levels in the lung for a longer period. In fact, 3 h after pulmonary administration, the amount of FTZ was 11 µg per g of organ, whereas for the other formulations, the respective value ranged from 1 to 7 µg per g of organ. In the liver and in the spleen, the amount of FTZ was very similar for all analyzed formulations with the exception of FTZ HP-γ-CyD that presented the highest and the lowest values in the spleen and in the liver, respectively, 0.5 and 3 h after inhalation. The inhalation of FTZ γ-CyD resulted in the lowest FTZ values in the lung and concomitantly the higher values in the blood for the analyzed periods. Overall, the FTZ HP-γ-CyD complex seems to be the best formulation for delivering this glucocorticoid to the lungs. 

As summary, a comparison for the two delivery systems, liposomes and CyD complexes using the i.n. and pulmonary routes, respectively, was performed and the amount of FTZ (µg/g lung) 0.5 and 3 h after administration is shown in Table 5.

The results obtained clearly demonstrate that the instillation of FTZ liposomes allowed achieving the highest levels of the glucocorticoid in the lungs. Taking into account that the dose administered i.n. was 1 mg/kg for a mouse with a body weight of 25 g weight means that 25 µg of FTZ was the instilled amount. In addition, the i.n. administration allowed knowing exactly the dose that each animal receives. The pulmonary route only permits knowing an approximate value of the administered dose. Nevertheless, an in vitro assay was performed (data not shown) to evaluate the experimental conditions of the inhalation chamber before performing the in vivo studies. The same amount of the tested FTZ dry powders was inserted in the donor compartment and the openings at the tapered ends of the animal holders of the inhalation chamber were plugged with cotton. The air pressure was applied at the same experimental conditions as those used in in vivo studies (0.4 kg/cm^2^ for 5 min). After that, the cottons were collected, extracted with ethanol, and FTZ content was determined. Taking into consideration the amount of FTZ incorporated in CyD complexes (Table 4), the amount of drug recovered in all ports ranged from 10 to 15% of the total FTZ. In addition, no statistically significant differences were observed for the 6 ports of the inhalation chamber (*p* < 0.05). Moreover, as 200 mg of each CyD FTZ formulation was inserted in the donor compartment, this means that an average amount of FTZ ranging from 500 to 600 µg reached the orifices of the animal holder tubes. Nevertheless, this does not mean that each animal received the above referred amount of FTZ. We can only say that mice were exposed to 500 to 600 µg of FTZ and that the dose was below 20 mg/kg of body weight. 

#### 3.4.2. Establishment of the Murine Asthma Model

The establishment of a murine model of asthma is essential for testing the safety and therapeutic efficacy of developed formulations. Although a high variety of animal species has been used, mice constitute the most commonly species used. In the present work, for inducing the allergic airway, the initial sensitization was performed by i.p. injections of OVA, in combination with the adjuvant aluminum hydroxide, while the challenge was performed using i.n. instillation of OVA [24,25]. 

**Figure 7:**
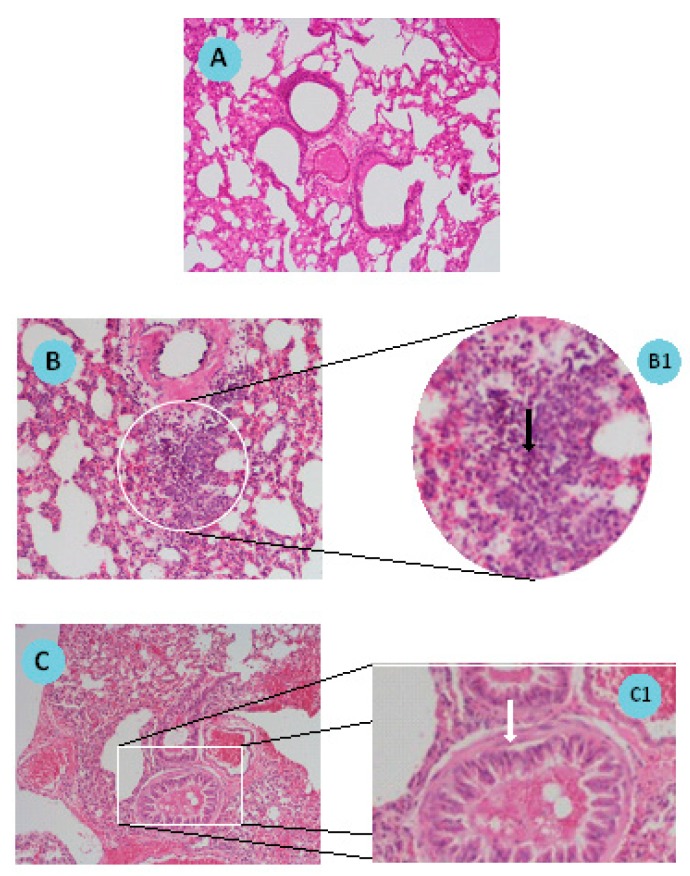
Sections of mouse lung (**A**) non-induced (Control); (**B**,**C**) chicken ovalbumin (OVA)/Aluminum sensitized/challenged. B1 and C1 are close-up images of B and C sections, respectively. Tissue sections were stained by hematoxylin-eosin standard procedure and observed by microscopy (magnification: A, B and C: 100×; B1 and C1: 400×). B1 black arrow indicates neutrophil infiltration; C1: White arrow indicates smooth muscle layer hyperplasia.

Histology and microscopic analysis were assayed to evaluate the establishment of the murine asthma. This analysis allowed the visualization of alterations in lung tissues in comparison with naïve mice. In Figure 7, lung sections of control mice and animals submitted to sensitization/challenge with OVA are shown. 

Figure 7A presents a lung section where no microscopic changes are observable, as expected for a lung from a naïve animal. Figure 7B,C represent two aspects of the development of asthma in mice. Figure 7B shows cellular infiltration with an increased number of eosinophils in a lung section of an induced animal. The respective enlarged image (Figure 7B1) shows the eosinophils pointed with the black arrow. Figure 7C shows bronchioles with hyperplasia of the smooth muscle layer. In Figure 7C1, muscle hyperplasia is signed by a white arrow. Histologic examination of haematoxylin and eosin stained lung specimens of induced animals confirmed the establishment of the asthma model in mice.

## 4. Discussion

FTZ is often prescribed as a first line-therapy for the effective management of pulmonary diseases such as asthma and rhinitis. Drug delivery systems, in particular liposomes and CyD complexes, are able to change in vivo biodistribution of incorporated molecules and have received great attention in recent years [11,12,13]. In this work, FTZ was used as a model drug to study the biopharmaceutical advantages of its incorporation into two different systems: The incorporation of FTZ in liposomes for i.n. administration, and the association to CyD complexes, aiming to improve the in vivo profile of FTZ following pulmonary administration of resultant dry powders. 

When designing FTZ liposomal formulations, it is crucial to achieve high loadings for this glucocorticoid; otherwise a high amount of lipid would be necessary to reach the desirable therapeutic dose. To achieve this goal, a combination of several factors, such as lipid composition, physicochemical properties of the drug, and liposomes preparation method should be considered. The DRV method used for incorporating FTZ in liposomes was selected, as it allows obtaining higher incorporation efficiencies than the simple thin-film hydration method [10,26,29]. The preparation of FTZ liposomes with neutral, negative, and positively charged lipid compositions was performed aiming to study their influence on FTZ incorporation parameters and to select the best lipid mixtures. Due to its hydrophobic character, FTZ is assumed to be incorporated in the lipid bilayer. The main phospholipids used, PC and PG, were selected on the basis of their low *T*c (below 0 °C), meaning that at room temperature phospholipids are in liquid-crystalline phase, lipid bilayers are in a less ordered structure, and the chance of FTZ to be incorporated is higher [31]. In fact, neutral and negatively charged liposomes prepared with PC or PC:PG, respectively, resulted in the lipid nanoformulations with higher FTZ incorporation parameters. The reduction on incorporation parameters of FTZ liposomes with PC and CHOL is based on the fact that CHOL is also inserted within lipid bilayers competing with the accommodation of the glucocorticoid. Nevertheless, for all tested FTZ nanformulations, liposomes presented small mean size values and homogeneous size distribution: Mean size ranged from 0.16 to 0.21 µm and PdI was below 0.2. 

Another strategy to solve low solubility of FTZ or deficient accumulation in the lungs after inhalation involves the use of CyDs, supramolecular structures able to include water-insoluble molecules [32]. CyDs may increase drug stability and bioavailability at the drug target site. In this sense, drug association to CyDs complexes can also increase drug action duration as drug complexes might promote a prolonged and controlled release of the incorporated material. There are a variety of CyDs derivatives in the market obtained by chemical synthesis, exhibiting different characteristics such as solubility, stability, and volume of the hydrophobic cavity, allowing controlling the activity and entrapment ability of incorporated drugs. In previous work [17], the influence of different CyDs on physical characteristics of FTZ CyDs inhalation dry powders was evaluated. The best characteristics for pulmonary delivery were obtained with FTZ Ac-γ-CyD complex that permitted FTZ to reach the lungs in a higher percentage in relation to the emitted dose [17].

The in vitro experiments developed in the present work aimed to investigate the potential toxic effect of FTZ formulations in the presence of a respiratory cell line, the A549. This cell line is useful for studying the mechanisms of drug delivery via the pulmonary route and in examining the transport of low molecular weight drugs and xenobiotic across the surface of the alveoli [33]. The in vitro tests demonstrated that FTZ in the free form or incorporated in neutral, positive, and negatively charged liposomes did not elicit toxic effects towards the A549 cell line, as cellular viability was always superior to 90% for all tested formulations. Regarding FTZ CyD complexes, and according to the in vitro results, neither FTZ CyD formulations nor empty CyDs or FTZ in the free form resulted in significant reduction on cellular viability of A549 cells. Overall, the in vitro tests demonstrated that both drug delivery systems used for incorporating FTZ are safe to be applied in vivo.

The in vivo biodistribution profile of FTZ in the liposomal form after i.n. administration and in the form of FTZ CyD complexes after pulmonary delivery was assessed and compared with the biodistribution profile of the drug free form. Particular interest was taken on the amount of FTZ accumulated in lungs. The obtained results showed that FTZ incorporated in liposomes resulted in much higher levels of this glucocorticoid in the lungs in comparison with free FTZ: 20 and 30 times, at 0.5 and 3 h after i.n. administration, respectively. Another important result was the determination of FTZ in blood as it represents the amount of drug that has been delivered and absorbed systemically from the lungs [34]. The amount of FTZ observed for mice that received FTZ in the free form, 30 min after instillation, proves that FTZ reached the lungs. The i.n. administration of FTZ liposomes led to a constant amount of the corticosteroid in the blood for 3 h (around 4 µg), whereas a reduction after the instillation of the free FTZ was observed from 6 to less than 2 µg during the same analyzed time. The results obtained led us to conclude that FTZ in liposomal form was able to promote a depot in the lungs. 

In the case of pulmonary administration of FTZ CyD complexes, the design of a nose-only inhalation chamber (Figure 1) was crucial to perform the biodistribution studies [19]. The ability of each CyD to deliver FTZ in the organs was evaluated and the results have demonstrated that for FTZ associated to HP-CyD complex, a higher amount of FTZ was obtained in the lung in comparison with the respective free form. This achievement was observed 0.5 and 3 h after animals were exposed to dry powders of FTZ CyD complexes for 5 min. Three hours after exposure, a slight decrease in the FTZ value in the lung was observed compared with the results for the 0.5 h time point. This could be an advantageous outcome as long as a sustained release of the glucocorticoid in the lung is a desirable result. Curiously, the already mentioned study of aerodynamic properties of inhaled particles containing FTZ CyDs indicates FTZ acetyl-γ-CyD complex as having the best characteristics for pulmonary delivery [17]. The results obtained in vivo presented in this study do not corroborate such early in vitro findings. In fact, in vitro methods could only predict in vivo behavior, but do not replace animal experiments. For this reason, the possibility of testing developed formulations in an animal model of disease is of major relevance, and a reason why a mice model of asthma was established. Mice constitutes the most common species used to model allergic airway inflammation mainly for practical reasons, including relatively low handling costs and reduced amount of formulations needed. However, as mice do not spontaneously develop asthma, an artificial asthmatic reaction had to be induced in the airways. Several mouse models of acute allergic pulmonary inflammation are described in the literature. Different protocols for sensitization, use an allergen, in most of the cases OVA, in combination with an adjuvant, administered by the i.p. route, followed by a challenge of the allergen. In the late steps, the allergen may be inhaled as a nebulized formulation, or administered by intratracheal or i.n. instillation of an aqueous formulation [35]. In the present work, the sensitization was performed by i.p. injections of OVA in combination with the adjuvant aluminum hydroxide, while the challenge was done using i.n. instillation of OVA in saline, according to Henderson et al. [24] and Oliveira et al. [25].

Histological analysis of lung sections of sensitized/challenged mice is a valuable tool to validate the establishment of the pathology. Indeed, in the present work, the visualization of lung sections after hematoxylin and eosin staining confirmed the cellular infiltration with an increased number of eosinophils and bronchioles with hyperplasia of the smooth muscle when compared to naïve mice, these observations being in accordance with the literature [24,25,35].

## 5. Conclusions

In this study, FTZ was used as a model drug incorporated in two different delivery systems: Liposomes and CyD complexes. The in vitro studies performed in the presence of a respiratory cell line demonstrated the safety of the so-developed FTZ liposomes, as well as FTZ CyD complexes previously developed. The benefits in terms of biodistribution profile of this compound following their association to liposomes or CyD complexes was evaluated using two different non-invasive routes, instillation and pulmonary delivery, respectively. The in vivo studies performed after instillation showed an advantage regarding the use of FTZ in the liposomal form as an increase of the glucocorticoid in lungs was observed (ranging from 10 to 30 times) in comparison to the FTZ in the free form. FTZ CyD complexes besides presenting in vivo lower accumulation levels for this glucocorticoid constitute another valuable approach for pulmonary delivery as dry powders for asthma management. Nevertheless, the pulmonary administration of other delivery systems with different mean aerodynamic properties may result in better biodistribution profiles. In addition, the strategy followed in the present work can be applied for other molecules. The establishment of the murine model of asthma was an important issue achieved in the present work. The therapeutic evaluation of both FTZ delivery systems, in the murine asthma model, should be considered in further studies.

## Figures and Tables

**Table 1:** Physical properties of fluticasone propionate (FTZ).

**Chemical structure**	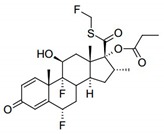
**Molecular weight**	500.57 g/mol
**LogP**	3.4
**Solubility in water**	0.51 mg/L

Data obtained from http://www.drugbank.ca/drugs/DB00588, accessed at June 2019 [20].

**Table 2:** Physicochemical Properties of FTZ Liposomes.

Nanoformulation N°	Lipid Composition (Molar Ratio)	(FTZ/Lip)_i_ (µg/µmol)	(FTZ/Lip)_f_ (µg/µmol)	I.E. (%)	Size (µm) (PdI)	Zeta Pot. (mV)
N1	PC	25 ± 2	7 ± 0.2	27 ± 1	0.19 (<0.2)	−2 ± 1
N2	PC: CHOL (2:1)	26 ± 1	1 ± 0.1	2 ± 1	0.21 (<0.2)	−3 ± 1
N3	PC: SA (9.5:0.5)	30 ± 7	3 ± 0.5	10 ± 2	0.20 (<0.2)	+5 ± 1
N4	PC: PG (7:3)	16 ± 2	4 ± 0.4	26 ± 5	0.16 (<0.2)	−25 ± 2

Initial lipid concentration (Lip)_i_—20 μmol/mL; Initial FTZ concentration (FTZ)_i_—500 µg/mL; (FTZ/Lip)_i_: Initial FTZ to lipid ratio; (FTZ/Lip)_f_: Final FTZ to lipid ratio; I.E. (%)—Incorporation efficiency = [(FTZ/Lip)_f_]/[(FTZ/Lip)_i_] × 100; Size—mean size of liposomes; PdI.—polydispersity index; Zeta Pot.—Zeta Potential; PC—phosphatidyl choline; PG—phosphatidyl glycerol; CHOL—cholesterol; SA—stearylamine.

**Table 3:** Physical properties of cyclodextrin (CyDs) used and mass median aerodynamic diameter (MMAD) of the FTZ CyD complexes *.

Cyclodextrin	Acetyl-γ-CyD	HP-γ-CyD	γ-CyD
Chemical structure	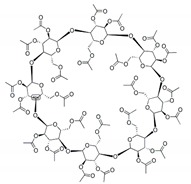	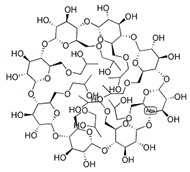	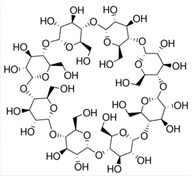
MW (g/mol)	2306.01	1761.76	1297.12
MMAD FTZ CyD complexes (µm) *	2.04 ± 0.18	2.32 ± 0.07	2.07 ± 0.11
d5 (µm) *	0.96	1.17	1.13
d50 (µm) *	2.01	2.07	1.81
d95 (µm) *	6.24	6.81	5.98

* data previously published in Drumond et al. (2014) [17].

**Table 4:** Quantification of FTZ incorporated in CyD complexes.

FTZ CyD Complexes *	Amount of FTZ (µg)
FTZ Acetyl-CyD	816
FTZ HP-CyD	977
FTZ γ-CyD	740

* 5 mg of the FTZ CyD complexes dry powders were used.

**Table 5:** Comparison of administration routes for FTZ: Pulmonary vs. i.n.

Formulation	µg/g Lung 0.5 h	µg/g Lung 3 h
pulmonary delivery		
Free FTZ	8.4 ± 1.4	7.6 ± 0.3
HP-CyD FTZ	11.1 ± 1.8	9.8 ± 1.6
intranasal delivery		
Free FTZ	3.0 ± 0.2	0.8 ± 0.3
LIP FTZ	56.7 ± 7.7	27.6 ± 5.3
